# Erratum: Synergistic effects of ion transporter and MAP kinase pathway inhibitors in melanoma

**DOI:** 10.1038/ncomms13080

**Published:** 2016-09-29

**Authors:** Ugur Eskiocak, Vijayashree Ramesh, Jennifer G. Gill, Zhiyu Zhao, Stacy W. Yuan, Meng Wang, Travis Vandergriff, Mark Shackleton, Elsa Quintana, Arthur E. Frankel, Timothy M. Johnson, Ralph J. DeBerardinis, Sean J. Morrison

Nature Communications 7 Article number:12336 10.1038/ncomms12336 (2016); Published 08 22 2016; Updated 09 29 2016.

Arthur E. Frankel, who helped to interpret the clinical relevance of the findings, was inadvertently omitted from the authors list during the final stages of production. This has now been corrected in both the PDF and HTML versions of the Article.

