# Supplementary material for: Synergistic effects of ion transporter and MAP kinase pathway inhibitors in melanoma
Source: Nat Commun. 2016 Aug 22;7:12336. doi: 10.1038/ncomms12336 (PMC4996948; doi:10.1038/ncomms12336)
Supplement: Supplementary Information — Supplementary Figures 1-8 [file ncomms12336-s1.pdf]

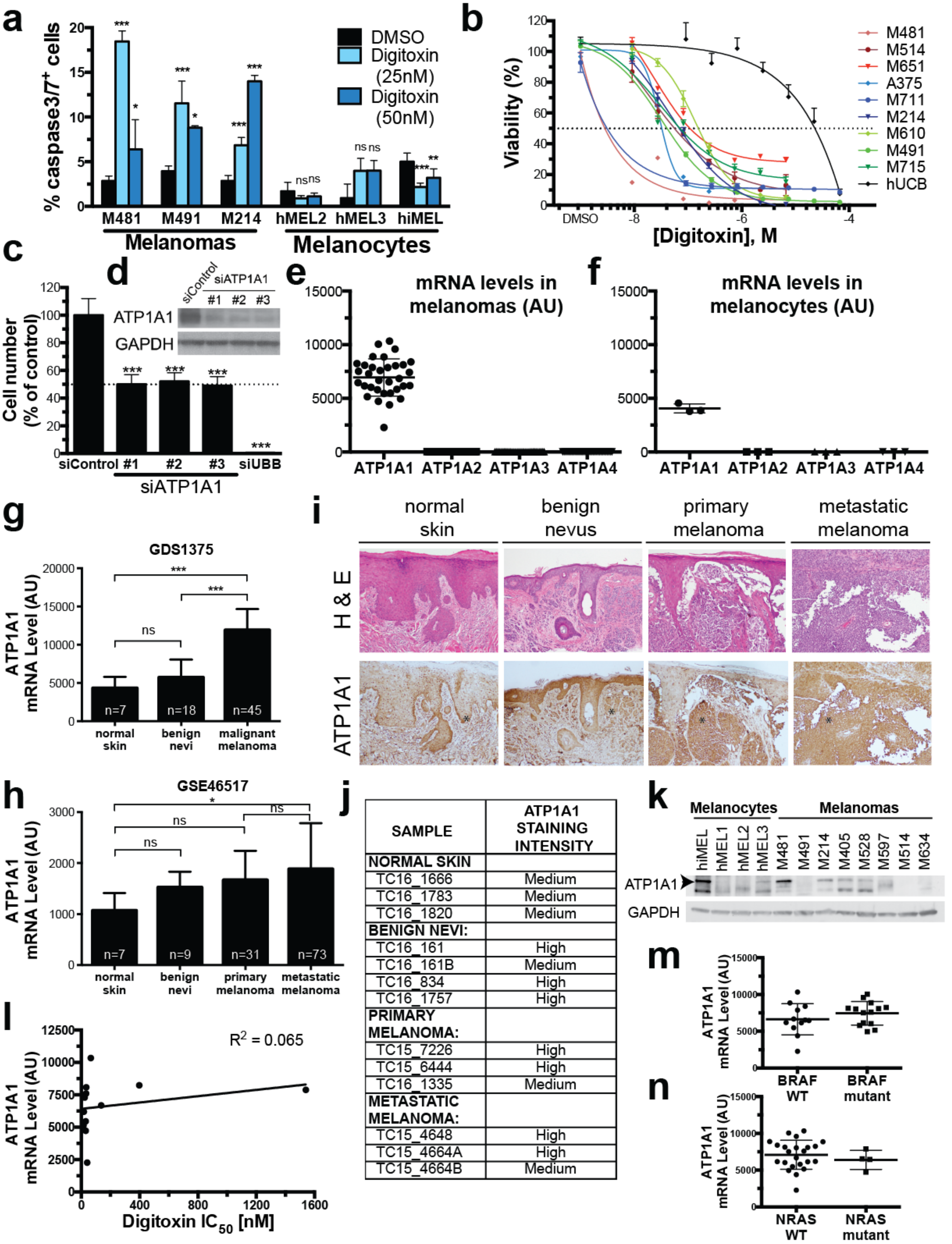

SAMPLE	ATP1A1 STAINING INTENSITY
NORMAL SKIN	
TC16_1666	Medium
TC16_1783	Medium
TC16_1820	Medium
BENIGN NEVI:	
TC16_161	High
TC16_161B	Medium
TC16_834	High
TC16_1757	High
PRIMARY MELANOMA:	
TC15_7226	High
TC15_6444	High
TC16_1335	Medium
METASTATIC MELANOMA:	
TC15_4648	High
TC15_4664A	High
TC15_4664B	Medium

Supplementary Figure 1: Digitoxin induces apoptosis in primary human melanoma cells but not in normal melanocytes, which express lower levels of the cardiac glycoside target, ATP1A1. (**a**) The percentage of caspase-3/7$^+$ apoptotic cells among melanoma cells cultured from three patients (M481, M491, and M214) or normal melanocytes cultured from two donors (hMEL2 and hMEL3) or immortalized melanocytes (hiMEL) treated with 25 nM or 50 nM digitoxin for 16 hours (n=3 replicate cultures for each cell type tested in the same experiment). Statistical significance was assessed by one-way ANOVA followed by Dunnett's multiple comparisons test (**P*<0.05; ***P*<0.01; ****P*<0.001). (**b**) Dose response curves of cultured A375 melanoma cells, primary umbilical cord blood cells (hUBC), and melanomas from 8 patients that exhibited sensitivity to digitoxin in culture. (**c**) Number of A375 cells 4 days after transfection with 3 different siRNAs against *ATP1A1*. Statistical significance was assessed by one-way ANOVA followed by Dunnett's multiple comparisons test (****P*<0.001; n=7 replicate cultures from 2 independent experiments). Controls included siRNAs against *LON Peptidase N-Terminal Domain And Ring Finger 1* (*LONRF1*; siControl; a negative control that is generally not required by cells) and Ubiquitin B (UBB, a positive control that is generally required by cells). (**d**) ATP1A1 protein levels by western blotting of A375 cells 72h after siRNA transfections. (**e-f**) Microarray analysis of transcript levels for the Na^+/K^+ ATPase alpha subunits *ATP1A1*, *ATP1A2*, *ATP1A3*, and *ATP1A4* in melanomas obtained from 33 patients (**e**) and in melanocytes from 3 donors (**f**, note that the *ATP1A1* data in panels e and f are the same as in Figure 1e). (**g**) Comparison of *ATP1A1* mRNA levels by microarray analysis in normal skin, benign nevi, and melanomas from GDS1375[35] in the NCBI GEO database. (**h**) Comparison of *ATP1A1* mRNA levels by microarray analysis in normal skin, benign nevi, primary melanoma, and metastatic melanoma from GSE46517[36] in the NCBI GEO database. (**i**) Representative images of ATP1A1 immunohistochemistry on paraffin sections from normal human skin, a benign nevus, primary melanoma, and metastatic melanoma. Asterisks (*) highlight key areas of ATP1A1 expression. (**j**) Table summarizing ATP1A1 expression by immunohistochemistry. The melanocytic portions of the tissue were assessed for ATP1A1 staining intensity and scored as "none", "low", "medium", or "high" by a dermatopathologist. (**k**) ATP1A1 protein levels by western blotting of 3 melanocytes (hMEL1, hMEL2 and hMEL3), immortalized melanocytes (hiMEL) and 8 melanomas (M481, M491, M214, M405, M528, M597, M514 and M634). (**l**) Lack of correlation between the sensitivity of melanoma lines to digitoxin in culture and *ATP1A1* transcript levels. (**m-n**) *ATP1A1* transcript levels in *BRAF* mutant versus *BRAF* wild-type melanomas (**m**) and in *NRAS* mutant versus *NRAS* wild-type melanomas (**n**) were not significantly different (Unpaired student's t-test). The Cancer Genome Atlas (TCGA) RNAseq data (https://tcga-data.nci.nih.gov/tcga) also show no difference in *ATP1A1* expression between primary and metastatic melanoma.

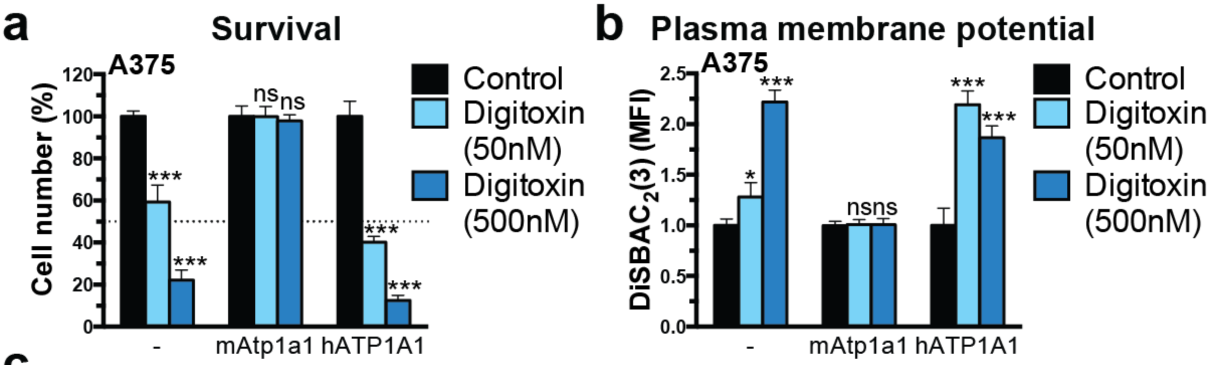

c

melanoma ID	AT MELANOMA BANKING								AFTER MELANOMA BANKING			
	age/ sex	AJCC clinical stage	tumor site	prognostic information				therapy before banking	therapy after banking	sites of metastasis	survival (months after banking)	AJCC at death or at present
				primary thickness (mm)	primary tumor ulceration	Regional LN (#+)	visceral disease detected by scan? Y/N					
**481**	67/M	IIIB	Subcutaneous	0.5mm (resected - 11y.)	Y	-	N	surgery	whole brain radiation, bortezomib	brain, lung, heart, mesentery, omentum, retroperitoneum, subcutaneous tissues and left gluteus maximus muscle metastases	14.7	IV
**491**	41/F	IIIC	Lymph Node	2.75mm (resected - 18mo)	Y	-	N	surgery	IFN	subcutaneous nodules recurrence. Progressive soft tissue and distant LN disease	19.2	IV
**214**	51/M	IIIC	Lymph Node	3.7mm (resected- 1y)	N	10	N	surgery	IFN, whole brain radiation	local/regional nodal recurrence, adrenal and brain metastasis	7.7	IV
**610**	76/F	IIA	Primary Cutaneous	3.5mm	N	-	-	none	unknown	unknown	8.9 (death unrelated to melanoma	at least IIA
**487**	54/M	IV	Cutaneous	9.0mm (resected - 7mo.)	Y	1	Y-brain, lung, liver, muscle	surgery, bortezomib, IFN	chemotherapy	extensive local recurrence, lung, brain, liver, gallbladder, left gluteus maximus muscle metastases	6.6	IV
**514**	49/M	IIIC	Lymph Node	concurrent 16.0mm	Y	2	N	none	none	multiple cutaneous/soft tissue metastasis, lung	3.4	IV
**651**	39/F	IIIB	Lymph Node	0.76mm (resected - 22mo.)	N	1	N	surgery	none	none	>22	IIIB
**711**	73/M	IIIC	Subcutaneous	1.32mm	N	0	Y	surgery	none	lung	15	IV
**715**	81/F	IV	Subcutaneous	3.48mm	N	0	Y	surgery	radiation	neck	9	IV
**UM12**	75/M	IIB	Lymph Node	10mm	Y	14		none	unknown	unknown	6	unknown, deceased
**498**	83/M	IIIB	Primary Cutaneous	5.3mm	N	-	N	none	radiation	none	70	unknown, deceased
**499**			Cutaneous Satellite Metastasis									
**701**	76/M	IIIC	Subcutaneous	unknown primary		2	N	none	unknown	unknown	9.6 (death unrelated to melanoma	at least IIIC
**741**	69/F	IIIC	Lymph Node	9.58mm	Y	5	Y	none	radiation	brain and lungs	8.0	IV
**528**	72/M	IIIB	Lymph Node	unknown	-	1	lung masses (not melanoma)	none	chemotherapy	none	40.7*	IIIB for melanoma; IV for lung cancer
**405**	29/M	IIIB	Lymph Node	unknown	-	2	small brain nodule of uncertain etiology	none	IFN; IL-2	local/regional nodal recurrence, widespread soft tissue, cutaneous and mesenteric metastasis, lung, liver, spine, spleen, brain and pelvic metastases	12.4	IV
**634**	57/M	IIIC	Cutaneous Satellite Metastasis	3.8mm (resected - 7mo)	Y	1	lung nodules uncertain etiology	surgery	chemotherapy, radiation, Ipilimumab	brain and lung	25	IV
**660**	46/M	IIIC	Right neck lymph node	2.85	Y	multiple matted nodes positive	N	surgery	IFN	no further mets	58	IIIC

*patient died in hospice with metastatic lung cancer and advanced Chronic Obstructive Pulmonary Disease. There was no evidence of melanoma at time of death.

Supplementary Figure 2: The toxicity of digitoxin to melanoma cells in culture reflects an on-target effect mediated by ATP1A1. (**a-b**) Rescue of viability (**a**) and plasma membrane potential (**b**) by over-expression of the cardiac glycoside-insensitive mouse *Atp1a1*, but not human *ATP1A1*, in the A375 human melanoma cell line (n=3 replicate cultures per treatment. All data represent mean±s.d. Statistical significance was assessed by one-way ANOVA followed by Dunnett's multiple comparisons test for each treatment compared to controls (ns: not significant; *P<0.05; ***P<0.001). (**c**) Clinical characteristics of the patients from which the 17 melanomas used in this study were derived.

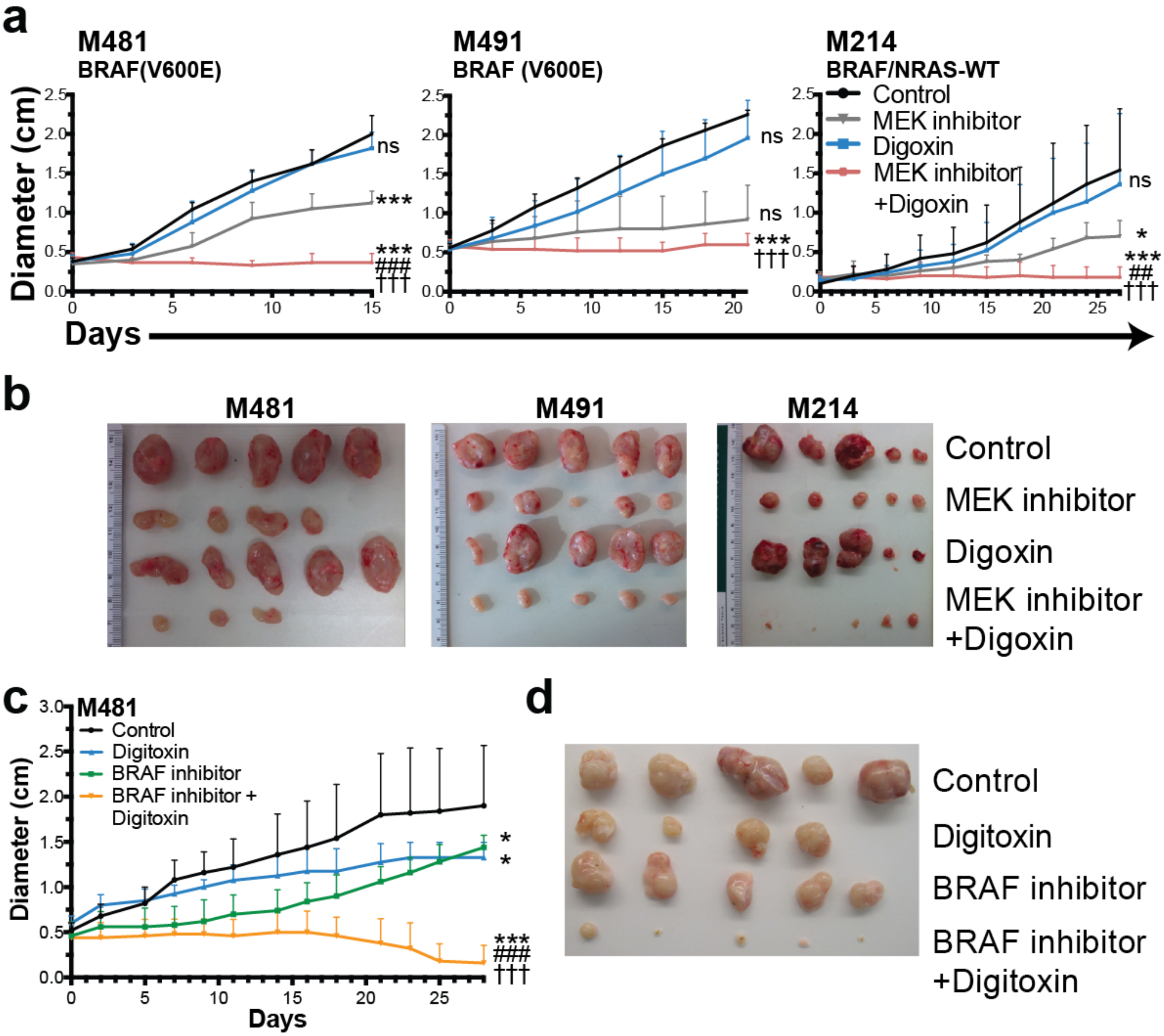

Supplementary Figure 3: Digoxin plus MEK inhibitor and digitoxin plus BRAF inhibitor additively or synergistically inhibit the growth of patient-derived xenografts. (**a-b**) NSG mice with subcutaneous xenografts derived from 3 different patients were treated with digoxin (10 mg/kg body mass/day) and/or MEK inhibitor (0.5 mg/kg body mass/day) to examine the effect on tumor diameter (mean±s.d.; n=3-5 mice per treatment per melanoma; each melanoma tested in an independent experiment). (**c-d**) M481 subcutaneous xenografts were treated with digitoxin (0.5 mg/kg body mass/day i.p.) and/or fed with BRAF inhibitor chow (417 mg/kg chow PLX4720) to examine the effect on tumor diameter (**c**) and size by photography (**d**). The statistical significance of each treatment compared to control (*, $P<0.05$; ***, $P<0.001$), combination treatment compared to MEK inhibitor alone (##, $P<0.01$; ###, $P<0.001$), or combination treatment compared to digoxin alone (†††, $P<0.001$) was assessed by two-way ANOVA followed by Dunnett's multiple comparisons test.

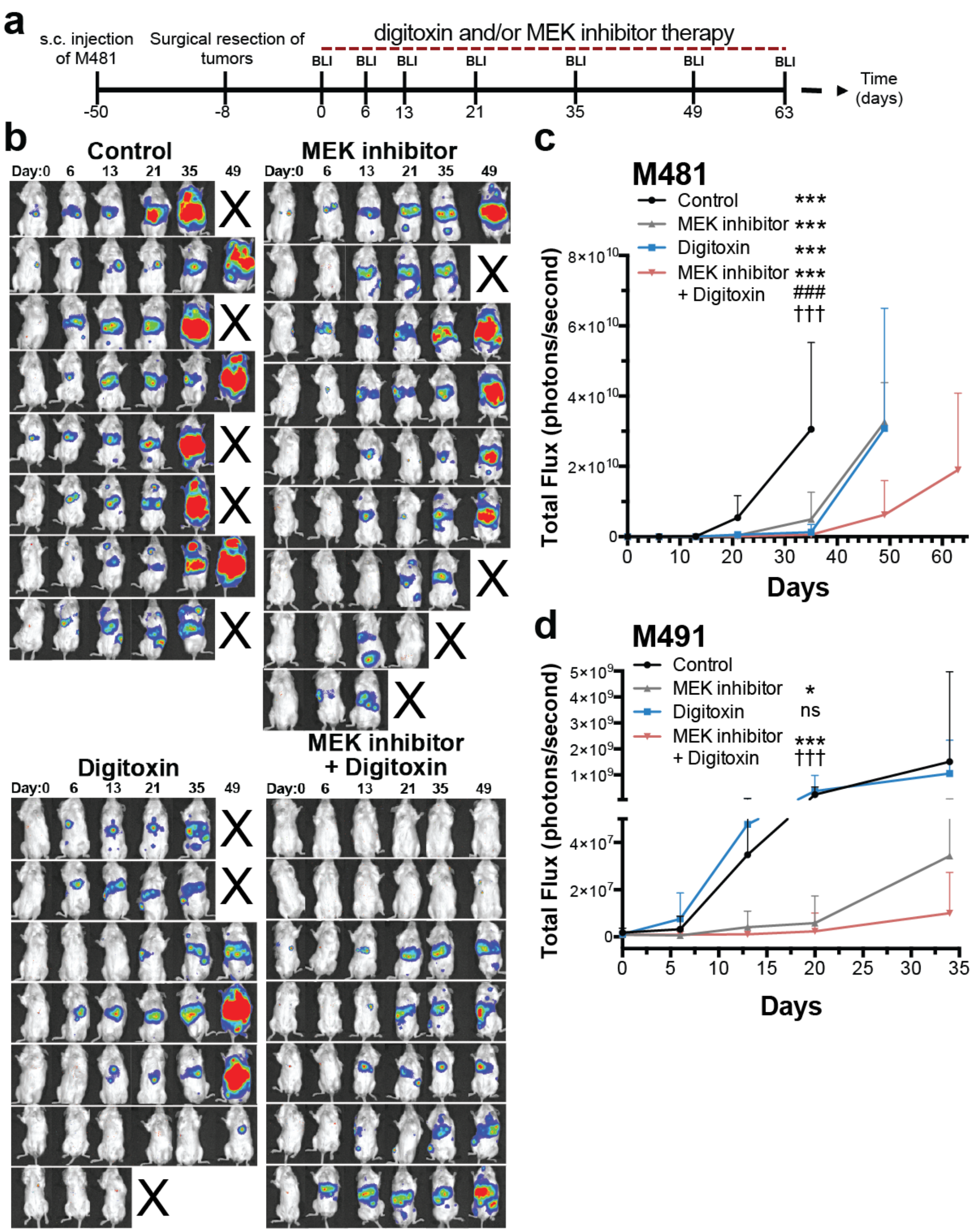

Supplementary Figure 4: Digitoxin plus MEK inhibitor additively or synergistically inhibits the growth of patient-derived xenografts and extends the survival of mice with metastatic human melanomas. (**a**) Outline of experimental plan (related to Figure 2b-d). (**b**) Bioluminescence (BLI) signal from all mice engrafted with luciferase-expressing melanomas derived from patient M481 after surgical excision of the primary subcutaneous tumor but before the onset of therapy (day 0) as well as 6, 13, 21, 35 or 49 days later. Missing images reflect mice that died before the indicated time points. (**c-d**) Total body bioluminescence signal (photons/s/cm^2/sr) from the mice in each treatment over time after the start of therapy: M481 (**c**, mean; n=5-8 mice/treatment as shown in panel b) and M491 (**d**, mean; n=4-8 mice/treatment as shown in panel b). Statistical significance was assessed by extra-sum-of-squares F tests followed by Bonferroni's multiple comparisons tests. Each treatment compared to control (ns: not significant; *, P<0.05; ***, P<0.001), or the combination compared to MEK inhibitor alone (###, P<0.001), or digitoxin alone (†††, P<0.001) are indicated. All data represent mean ± s.d.

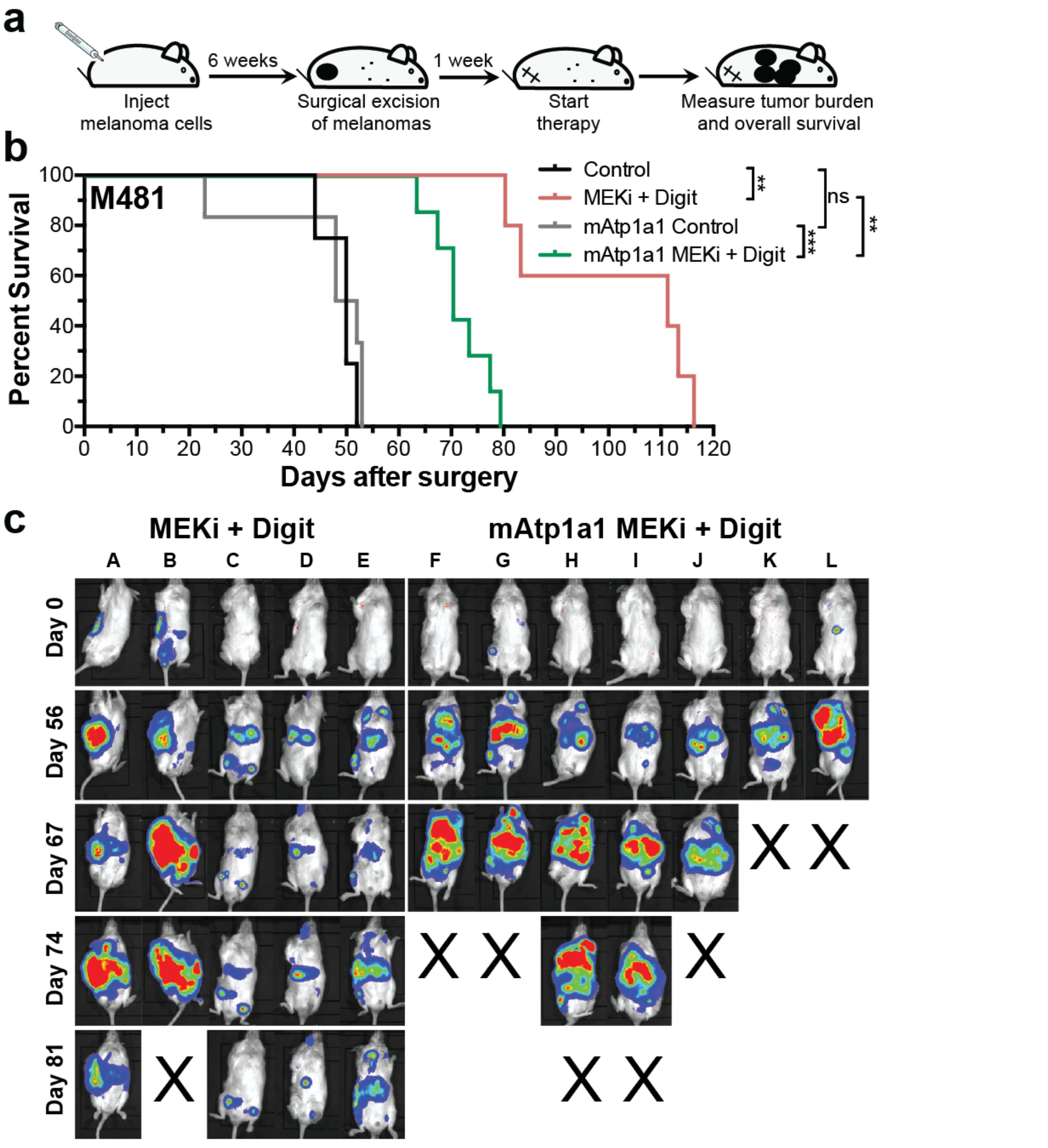

* mice with tumors at the site of surgery before starting therapy at day 0.

Supplementary Figure 5: Inhibition of ATP1A1 function is necessary for digitoxin plus MEK inhibitor to extend the survival of mice with metastatic human melanomas. (**a**) Outline of experimental plan. (**b**-**c**) Analysis of mice after surgical removal of primary subcutaneous melanomas, some of which over-expressed the cardiac glycoside-insensitive mouse m*Atp1a1* (n=4-7 mice/treatment). (**b**) Survival (not requiring euthanasia per animal care protocol) in days after surgery. Statistical significance was assessed by log-rank test followed by Bonferroni's multiple comparison's test (**, $P<0.01$; ***, $P<0.001$). (**c**) Bioluminescence images of mice treated with digitoxin plus MEK inhibitor in **b**.

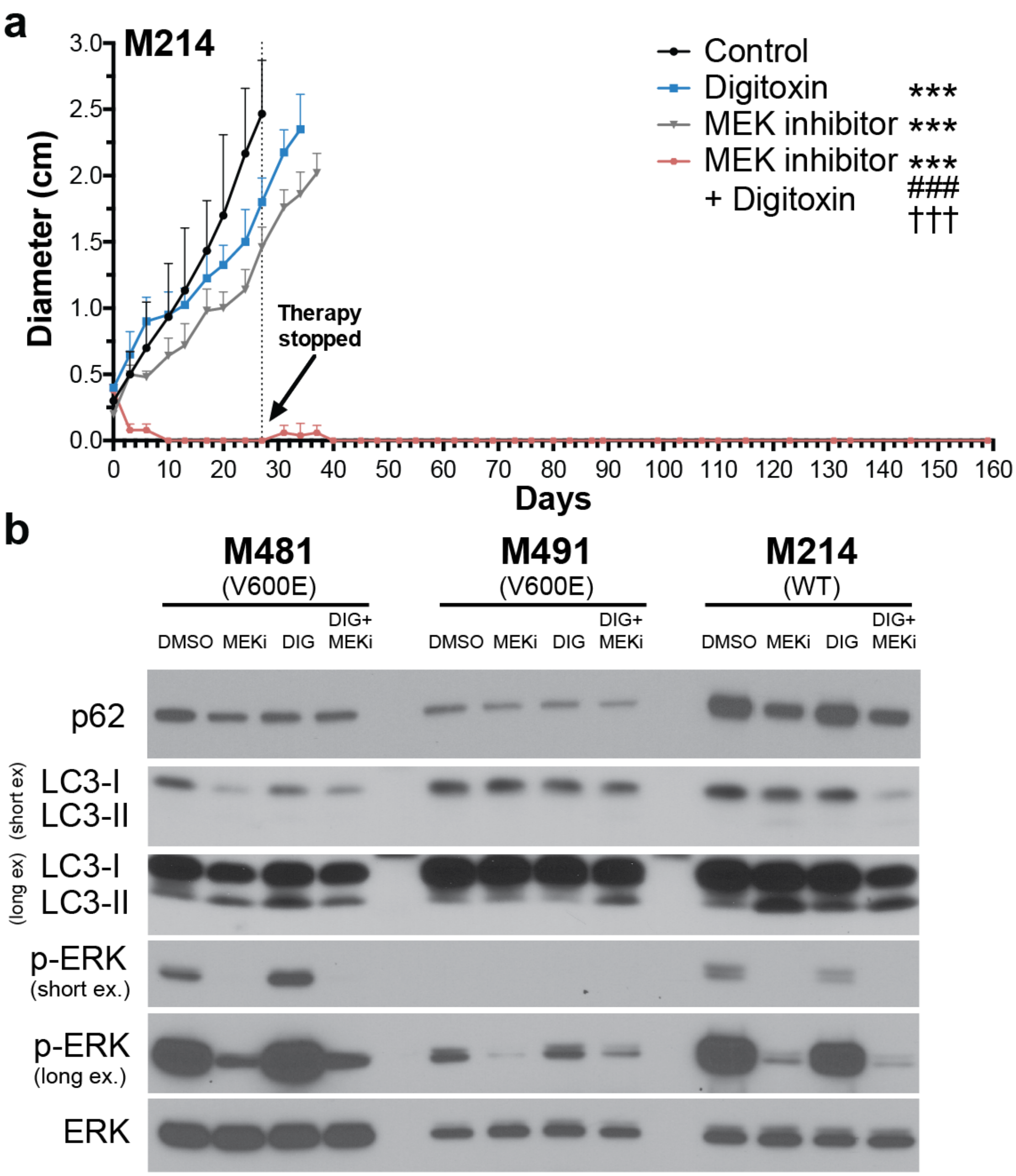

Supplementary Figure 6: Tumors that regressed to the point of not being palpable do not necessarily grow back after digitoxin plus MEK inhibitor treatment is stopped. (**a**) M214 subcutaneous xenografts were treated with digitoxin and/or MEK inhibitor (0.5 mg/kg body mass each by daily gavage) for 27 days then all therapy was stopped and tumor diameters were monitored until tumors reached 2 cm in diameter or for an additional 132 days in digitoxin plus MEK inhibitor treated mice (n= 3-5 mice per treatment). The statistical significance was assessed by log-rank test followed by Bonferroni's multiple comparison's test (each treatment compared to control (***, $P<0.001$) or the combination compared to MEK inhibitor alone (###, $P<0.001$), or digitoxin alone (†††, $P<0.001$)). Data represent mean ± s.d. (**b**) Western blot analyses of xenografted melanomas treated for 4 days in vivo with MEK inhibitor and/or digitoxin. Note that the pERK and ERK blots are the same as in Figure 3j (the data in Figure 3j were obtained in the same experiment as the data in panel b above).

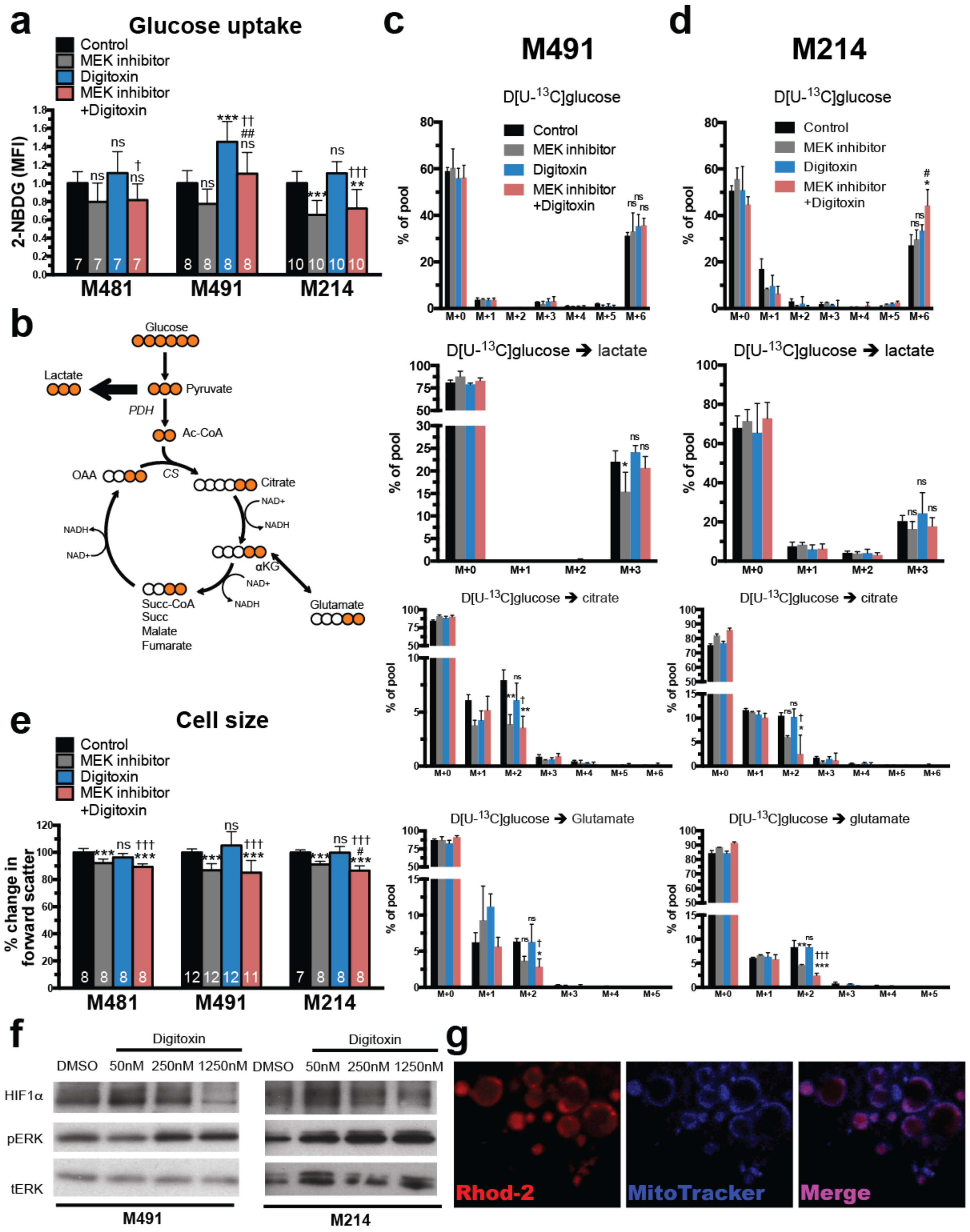

Supplementary Figure 7: MEK inhibitor, but not digitoxin, reduces glucose uptake and cell size in xenografted melanoma cells and in vivo isotope tracing is consistent with reduced glucose metabolism in the presence of MEK inhibitor. (**a**) Freshly dissociated cells from four day in vivo

treated melanoma xenografts were incubated with 2-NBDG (a fluorescent D-Glucose analogue) for 30 min at room temperature, washed and analyzed by flow cytometry (the number of mice per treatment is indicated in each bar; each melanoma was tested in 1-2 independent experiments). (**b**) Schematic of ^{13}C-labelled glucose metabolism. (**c-d**) Mice bearing melanomas from two patients (M491 (**c**) and M214 (**d**)) were treated for four days in vivo with digitoxin or MEK inhibitor or the combination. Sixty minutes after the last drug treatment mice were injected with a single bolus of ^{13}C-labeled glucose (2 g/kg body mass). After an additional 30 minutes, tumors were surgically excised, metabolites extracted, and analyzed by GC-MS (n=3-4 mice per treatment; each melanoma was tested in an independent experiment). (**e**) Cell size (forward scatter by flow cytometry) in live, freshly dissociated melanoma cells (the number of mice per treatment is indicated in each bar; each melanoma was tested in 2-3 independent experiments). (**f**) Western blot analyses of melanoma cells treated with DMSO or digitoxin for 8 hours in culture. (**g**) Representative images of A375 cells co-stained with rhod-2 and Mitotracker deep red to confirm mitochondrial localization of Rhod-2. In addition to the mitochondrial localization, Rhod-2 also localizes to nucleoli; however, nucleolar Rhod-2 signal is clearly distinguishable from mitochondrial Rhod-2 based on cellular morphology. We focused our analysis on mitochondrial Rhod-2 by drawing regions of interests around mitochondria and quantifying fluorescence only in these regions. For all panels, statistical significance was assessed by one-way ANOVA followed by Dunnett's multiple comparison's test except for M491 in **e** where where Levene's test indicated unequal variance among treatments. In that case, the Kruskal-Wallis test followed by Dunn's multiple comparisons test was applied. In all panels, each treatment compared to control (ns: not significant; *, $P<0.05$; **, $P<0.01$; ***, $P<0.001$), or the combination compared to MEK inhibitor alone (#, $P<0.05$; ##, $P<0.01$), or digitoxin alone (†, $P<0.05$; ††, $P<0.01$; †††, $P<0.001$). All data represent mean ± s.d.

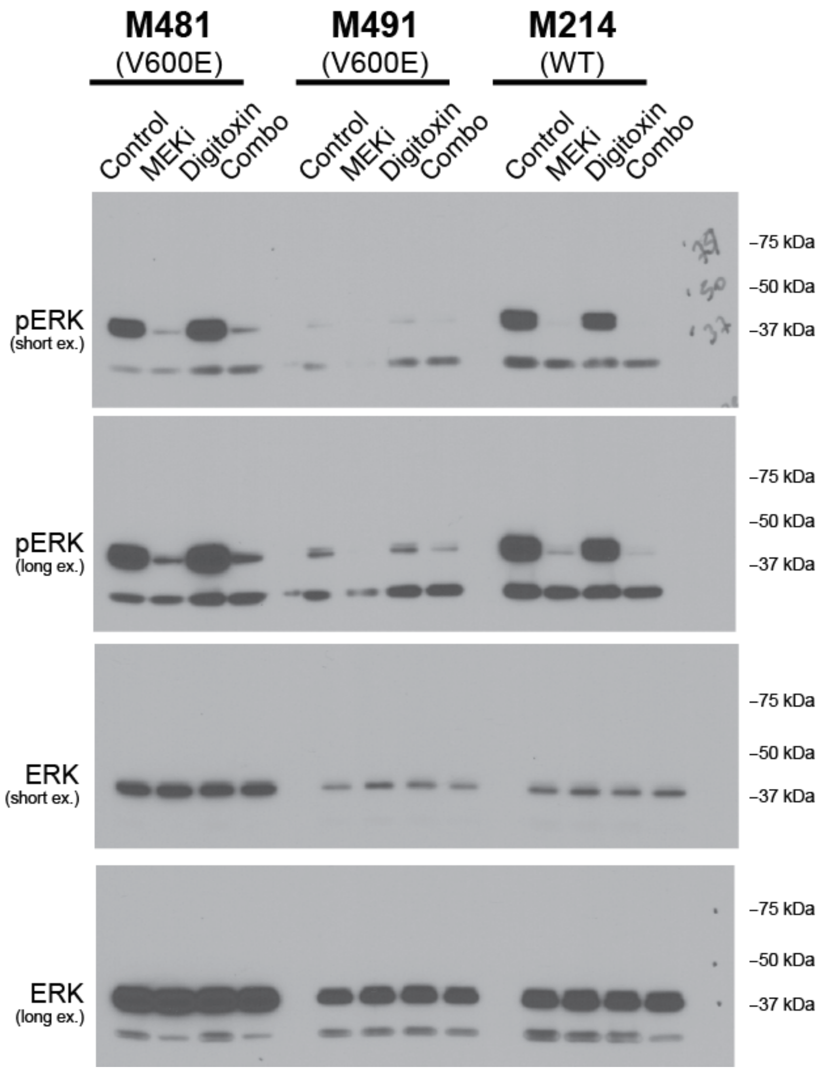

Supplementary Figure 8: **Full western blot images from Figure 3J**. Blots depict levels of phosphorylated or total ERK in xenografted melanoma cells treated for 4 days in vivo with MEK inhibitor and/or digitoxin.
